# Point-of-care ultrasound for perioperative deep vein thrombosis assessment in anesthesiology: a narrative review

**DOI:** 10.1016/j.bjane.2026.844793

**Published:** 2026-07-09

**Authors:** Luis Alberto Rodriguez Linares, Rodrigo Souza da Silva, Marco Antonio Duarte Carneiro

**Affiliations:** a Grupo de Apoio ao Adolescente e à Criança com Câncer (GRAAC), São Paulo, SP, Brazil; b Pontifícia Universidade Católica do Paraná (PUC-PR), Escola de Medicina, Curitiba, PR, Brazil; c Faculdade de Medicina da Universidade de São Paulo (FMUSP), São Paulo, SP, Brasil

*Dear Editor,*

Deep vein thrombosis remains a major cause of preventable perioperative morbidity, and pulmonary embolism is one of the most feared complications in surgical patients.^1^^,^^2^ Many episodes are clinically silent, which reinforces the importance of early recognition. Although duplex ultrasonography is the diagnostic reference standard, its availability may be limited during busy perioperative periods or in unstable patients who cannot be safely transported.^3^ These challenges have driven growing interest in point-of-care ultrasound as a rapid bedside method for identifying lower limb thrombosis. Anesthesiologists, who routinely use ultrasound for vascular access, regional anesthesia, and hemodynamic assessment, are well positioned to incorporate venous compression protocols into perioperative evaluation. This narrative review summarizes available evidence on the diagnostic accuracy, applicability, and practical considerations of bedside ultrasound for deep vein thrombosis assessment in the perioperative setting.

The evidence included prospective and observational studies evaluating two-point, three-point, or extended compression approaches performed by clinicians from anesthesiology, emergency medicine, intensive care, and hospital medicine. These studies compared bedside ultrasound with duplex ultrasonography or contrast venography. To provide context for the diagnostic performance estimates presented, studies were identified through a structured literature search of MEDLINE and Embase using terms related to point-of-care ultrasound, compression ultrasound, deep vein thrombosis, and perioperative care, supplemented by reference list review. The reported sensitivity and specificity ranges cited in this review derive primarily from published systematic reviews and meta-analyses rather than from original synthesis. The 26 representative studies informing this narrative review are listed in Supplementary Table S1.

Across 26 representative studies identified through this search, reported sensitivity for proximal deep vein thrombosis ranged from 90% to 96%, and specificity from 95% to 97%.^4^ These values suggest that trained clinicians may, under favorable conditions, produce diagnostic estimates in a range comparable to those reported in vascular laboratory settings; however, interpretation must remain cautious, as performance varies substantially with operator training, patient selection, disease prevalence, reference standard verification, and spectrum bias. The two-point protocol, which evaluates compressibility of the common femoral and popliteal veins, was the most frequently studied technique and proved efficient for rapid screening in low- to moderate-risk patients. The three-point method, adding the proximal femoral vein, and extended protocols that include more proximal or distal segments offered incremental diagnostic benefit in patients at higher risk or with suggestive clinical symptoms. No protocol reliably identified isolated distal calf thrombosis, and formal duplex scanning was recommended when distal disease was suspected.^5^ The main differences between two-point and three-point compression protocols are summarized in Table 1.

**Table 1:** Comparison between two-point and three-point compression ultrasound for deep vein thrombosis diagnosis.

Criteria	Two-point Compression Ultrasound (common femoral + popliteal veins)	Three-point Compression Ultrasound (adds femoral vein above the adductor canal)
Time and feasibility	Faster; ideal for bedside screening and emergency use.	Slightly longer exam; still feasible for perioperative assessment.
Overall diagnostic accuracy	High (sensitivity ≈ 90%–92%, specificity ≈ 95%–97%); may be considered for focused bedside assessment in selected perioperative patients with clinical suspicion of proximal DVT.	Similar overall accuracy; may detect additional cases of femoral thrombosis.
Detection of isolated femoral thrombosis	May miss up to 7% of isolated femoral thrombi.	Better sensitivity for isolated femoral segment thrombosis.
Distal DVT assessment	Does not evaluate calf veins.	Also does not assess calf veins; formal duplex ultrasonography required if suspicion remains high.
Learning curve	Shorter; easily standardized; ideal for training.	Slightly steeper; requires consistent identification of the femoral segment.
Best suited for	May be considered for focused bedside assessment in selected perioperative patients with clinical suspicion of proximal DVT; particularly useful for rapid pre-, intra-, and postoperative triage.	May provide a more extensive proximal venous assessment in high-risk or symptomatic patients; formal duplex ultrasonography remains indicated when available.
Impact on perioperative workflow	Maximizes agility and immediate decision-making (e.g., anticoagulation, regional block safety).	Maximizes completeness and safety in high-risk contexts.
Follow-up recommendation	Repeat ultrasound or full duplex ultrasonography at 5–7 days if clinical suspicion persists.	Same recommendation for high-risk patients with negative initial study.
Anesthesiology applicability	May support clinical assessment when proximal DVT is suspected before regional anesthesia or in intraoperative instability; findings should inform, not replace, clinical judgment and formal vascular imaging when available.	May provide a more extensive proximal venous assessment in high-risk or symptomatic perioperative patients; formal duplex ultrasonography remains indicated when available.

CUS, Compression Ultrasound; DUS, Duplex Ultrasonography; DVT, Deep Vein Thrombosis; POCUS, Point-of-Care Ultrasound.

The rapidity of point-of-care ultrasound is one of its most relevant advantages for anesthesiologists. Several studies have demonstrated that bedside evaluation shortens diagnostic delays compared with radiology-based imaging. Hospital-based cohorts reported average reductions of 5 to 6 hours in time to diagnosis, and emergency studies observed decreases exceeding one hour.^6^ In perioperative care, these time savings may affect anticoagulation decisions, scheduling of surgical procedures, and the safety of neuraxial anesthesia. Incidental detection of deep vein thrombosis during routine preoperative scanning, vascular access attempts, or ultrasound-guided regional blocks has been documented and may significantly alter perioperative management.^7^ Such findings highlight how venous evaluation can be seamlessly integrated into the ultrasound practices already common to anesthesiologists. It should be noted, however, that a positive proximal deep vein thrombosis finding may support clinical suspicion for pulmonary embolism, but a negative compression examination does not exclude pulmonary embolism; bedside ultrasound should be regarded as an adjunctive bedside tool rather than a surrogate for definitive vascular or pulmonary imaging.

The methodological limitations of the available evidence must be acknowledged. Many studies used convenience samples or included high-prevalence populations, which may overestimate accuracy. Blinding between bedside examiners and reference imaging results was not consistently reported. In some studies, negative bedside scans were not systematically verified by duplex ultrasonography, which introduces verification bias. Nonetheless, the consistency of diagnostic performance across heterogeneous study designs and operator backgrounds suggests that compression ultrasound is a useful bedside tool for proximal deep vein thrombosis identification in selected clinical scenarios.

For anesthesiologists, the implications extend across the perioperative continuum. In the preoperative setting, rapid bedside scanning can identify unrecognized venous thrombosis in patients undergoing orthopedic, oncologic, trauma, or other high-risk procedures. Detecting a proximal thrombosis may influence the selection and timing of neuraxial techniques and prompt adjustment of thromboprophylaxis. During intraoperative care, episodes of hypoxemia, hypotension, or acute right ventricular dysfunction may raise suspicion for pulmonary embolism. A focused femoral-popliteal compression ultrasound can assist in determining whether a deep vein thrombosis is likely, thereby supporting timely clinical decisions when diagnostic resources are limited.^8^ Although bedside ultrasound does not replace definitive imaging in unstable patients, it can guide early management while arrangements for confirmatory testing are underway.

In the postoperative period, targeted bedside evaluation may aid in the early detection of new or progressive thrombotic events in immobilized or oncologic patients. While routine screening is not supported by current evidence, selective examination in symptomatic or deteriorating patients is practical and may reduce diagnostic uncertainty. Postoperative compression ultrasound can be particularly useful in clinical contexts where transport to radiology carries risk or delay.^4^

Integration of point-of-care venous ultrasound into anesthesiology practice requires structured training and standardization. Most studies evaluating learning curves demonstrate that clinicians with existing ultrasound experience require relatively few supervised examinations to become proficient.^9^ The technique relies primarily on recognition of venous compressibility, a concept familiar to practitioners performing ultrasound-guided procedures. Incorporating venous scanning into perioperative ultrasound curricula and credentialing pathways can help ensure consistent performance. Standardizing scanning sequences, documentation, and image storage also facilitates quality assurance and interdisciplinary communication. Additional implementation considerations include the establishment of credentialing thresholds, protocols for image documentation and archiving, quality assurance processes, interdepartmental communication workflows, medicolegal accountability frameworks, and clearly defined thresholds for referral to confirmatory duplex ultrasonography.

Although the current body of evidence supports point-of-care ultrasound as a useful bedside technique, further investigation is needed to better define its role in perioperative outcomes. Few studies have focused specifically on anesthesiologists as primary operators, and the impact of bedside venous ultrasound on surgical timelines, regional anesthesia safety, or thromboembolism-related complications has not been formally quantified. Establishing competency benchmarks, validating risk-adapted scanning strategies, and exploring outcome-based applications are important next steps. Future research could also examine whether perioperative integration of compression ultrasound affects the timing of anticoagulation, length of hospital stay, or postoperative complication rates.

In summary, point-of-care venous ultrasound is an adjunctive bedside tool for selected perioperative scenarios, particularly when formal vascular imaging is delayed or unavailable. When performed by appropriately trained clinicians, compression ultrasound may provide useful bedside diagnostic support for proximal deep vein thrombosis assessment in these selected contexts. Applying a risk-adapted strategy ‒ using two-point compression ultrasound for general perioperative screening and reserving three-point or extended evaluations for high-risk or symptomatic patients ‒ balances efficiency with diagnostic precision. As ultrasound becomes increasingly embedded in perioperative care, venous compression protocols represent a natural extension of anesthesiologists' skill sets and a valuable addition to patient safety practices.

## Declaration of AI and AI-assisted technologies

During the preparation of this manuscript, the authors used Claude (Anthropic, claude.ai) and Paperpal solely for language editing and grammar checking. The authors reviewed and edited the output as needed and take full responsibility for the scientific content and final version of the manuscript.

## Data availability statement

Data not applicable. This study is a narrative review and did not generate or analyze original datasets.

## Note

A completed SANRA checklist is provided as Supplementary Material 1. The 26 representative studies informing this narrative review are listed in Supplementary Table S1.

## CRediT authorship contribution statement

**Luis Alberto Rodriguez Linares:** Conceptualization, Data curation, Formal analysis, Investigation, Project administration, Supervision, Writing – review & editing. **Rodrigo Souza da Silva:** Data curation, Formal analysis, Investigation, Methodology, Project administration, Visualization, Writing – original draft. **Marco Antonio Duarte Carneiro:** Data curation, Formal analysis, Methodology, Writing – review & editing.

## Conflicts of interest

The authors declare no conflicts of interest.

## Appendix Supplementary materials

Image, application 1

## Footnotes

Supplementary material associated with this article can be found in the online version at doi:10.1016/j.bjane.2026.844793.
