# Supplementary material for: Point-of-care ultrasound for perioperative deep vein thrombosis assessment in anesthesiology: a narrative review
Source: Braz J Anesthesiol. 2026 Jul 9;76(5):844793. doi: 10.1016/j.bjane.2026.844793 (PMC13425839; doi:10.1016/j.bjane.2026.844793)
Supplement: Supplementary file 1 [file mmc1.docx]

**BJAN-D-25-00810_Supplementary Material**

**Supplementary Table S1** Twenty-six representative studies informing this narrative review on point-of-care ultrasound for perioperative deep vein thrombosis assessment.

| **Study (Author, Year)** | **Country / Setting** | **Design / Population** | **Operator Specialty** | **CUS Protocol** | **Reference Standard** | **Principal Findings (Sn / Sp)** | **Limitations** |
| --- | --- | --- | --- | --- | --- | --- | --- |
| Lensing et al., 1989 | Netherlands / Vascular clinic | Prospective, 220 symptomatic outpatients | Vascular medicine | Two-point CUS | Contrast venography | Sn 100%, Sp 99% | Single referral centre; high-prevalence population |
| Cogo et al., 1998 | Italy / Outpatient | Prospective, 1,702 consecutive patients | Internal medicine | Two-point CUS | Venography (selective) | Sn 97%, Sp 98% | Algorithm study; symptomatic population only |
| Bernardi et al., 1998 | Italy / Outpatient | Prospective, 1,053 patients | Internal medicine / Radiology | Two-point CUS + D-dimer | Venography or follow-up | Sn 96%, Sp 98% | Not all negatives confirmed by venography |
| Birdwell et al., 1998 | USA / Academic hospital | Prospective, 445 symptomatic outpatients | Radiology | Duplex ultrasonography (reference) | Duplex ultrasonography | N/A – reference standard study | Selection bias; referral-based cohort |
| Wells et al., 2003 | Canada / Academic hospital | RCT/management, 1,096 patients | Internal medicine | Two-point CUS + Wells score + D-dimer | Venography | Sn 96%, Sp 97% | Management study, not pure diagnostic accuracy |
| Crisp et al., 2010 | USA / Emergency Department | Prospective, 47 patients | Emergency medicine | Two-point POCUS | Formal duplex ultrasonography | Sn 100%, Sp 99% | Small sample; single centre |
| Jang et al., 2010 | USA / Emergency Department | Prospective, 199 patients | Emergency medicine | Two-point POCUS | Formal duplex ultrasonography | Sn 95%, Sp 96% | Emergency-specific population; high prevalence |
| Kory et al., 2011 | USA / ICU | Prospective, 51 patients | Intensive care / Critical care | Two-point POCUS | Formal duplex ultrasonography | Sn 86%, Sp 96% | Small ICU sample; critically ill patients |
| Pomero et al., 2013 | Italy / Internal medicine wards | Meta-analysis, 16 studies | Non-radiologist clinicians | Two-point CUS | Duplex ultrasonography or venography | Sn 96%, Sp 97% (pooled) | Heterogeneous operator training; publication bias |
| Burnside et al., 2008 | USA / Emergency Department | Prospective, 105 patients | Emergency medicine | Two-point POCUS | Formal duplex ultrasonography | Sn 91%, Sp 98% | Single centre; convenience sample |
| Magazzini et al., 2007 | Italy / Emergency Department | Prospective, 132 patients | Emergency medicine | Three-point POCUS | Formal duplex ultrasonography | Sn 98%, Sp 95% | Single centre; unblinded outcomes |
| Frazee et al., 2015 | USA / Emergency Department | Prospective, 100 patients | Emergency medicine | Three-point POCUS | Formal duplex ultrasonography | Sn 100%, Sp 95% | Small sample; ED-specific population |
| Adhikari et al., 2015 | USA / Emergency Department | Prospective, 155 patients | Emergency medicine | Three-point POCUS | Formal duplex ultrasonography | Sn 100%, Sp 96% | Convenience sample; potential spectrum bias |
| Seyedhosseini et al., 2018 | Iran / Emergency Department | RCT, 130 patients (time-to-diagnosis) | Emergency medicine | Two-point POCUS | Formal duplex ultrasonography | Time reduction: > 1 hour | Not a diagnostic accuracy study; disposition focus |
| Abbasi et al., 2012 | USA / Emergency Department | Prospective, 61 patients | Emergency medicine | Two-point POCUS | Formal duplex ultrasonography | Sn 100%, Sp 98% | Very small sample; single institution |
| Mumoli et al., 2012 | Italy / Internal medicine | Prospective, 600 patients | Internal medicine | Simplified two-point CUS | Formal duplex ultrasonography | Sn 95%, Sp 97% | Non-blinded in 40% of cases |
| Blaivas & Lambert, 2004 | USA / Emergency Department | Prospective, 47 patients | Emergency medicine | Two-point POCUS | Formal duplex ultrasonography | Sn 100%, Sp 96% | Small sample; single site ED |
| Jacoby et al., 2007 | USA / Emergency Department | Prospective, 91 patients | Emergency medicine | Two-point POCUS | Formal duplex ultrasonography | Sn 89%, Sp 100% | Convenience sample; single centre |
| Fox et al., 2012 | USA / Emergency Department | Prospective, 47 patients | Emergency medicine | Three-point POCUS | Formal duplex ultrasonography | Sn 100%, Sp 100% | Very small sample; possible spectrum bias |
| Lee et al., 2019 | South Korea / ED (meta-analysis) | Meta-analysis, 18 studies (two-point vs. three-point) | Emergency medicine | Two-point vs. three-point CUS | Duplex ultrasonography | Two-point: Sn 94%, Sp 97%; Three-point: Sn 95%, Sp 96% | Heterogeneity across included studies |
| Zaki et al., 2024 | Multi-country / ED (systematic review) | Systematic review & meta-analysis, 21 studies | Emergency medicine / POCUS operators | POCUS compression techniques | Duplex ultrasonography or venography | Sn 95–96%, Sp 95–97% (pooled) | High heterogeneity; limited perioperative data |
| Sutin et al., 2005 | USA / Perioperative (case report/series) | Case series | Anesthesiology | Incidental two-point assessment during nerve block | Clinical + imaging confirmation | Proximal DVT detected incidentally | Very limited sample; case-series methodology |
| Kang et al., 2024 | South Korea / ED | Prospective learning curve study, 212 exams | Emergency medicine residents | Three-point POCUS | Formal duplex ultrasonography | Competency reached at 20–25 supervised exams | ED-focused; not perioperative context |
| Melo et al., 2025 | Brazil / ICU (systematic review) | Systematic review & meta-analysis, multi-organ POCUS for PE | Critical care / POCUS operators | Multi-organ POCUS including CUS | CT pulmonary angiography | DVT component: Sn 92%, Sp 94% | DVT data subset; not standalone CUS study |
| Colwell et al., 2010 | USA / Orthopedic surgical | Prospective, 1,000 elective arthroplasty patients | Orthopedic surgery / Radiology | Extended compression ultrasound | Duplex ultrasonography | Prevalence 1.1% asymptomatic DVT | Post-thromboprophylaxis cohort; low baseline prevalence |
| Wan et al., 2020 | China / ICU + surgical ward | Prospective, 320 high-risk surgical patients | Intensivist / Hospitalist | Three-point POCUS | Formal duplex ultrasonography | Sn 94%, Sp 97% | Asian population; high-risk surgical selection bias |

Note: Studies marked with * were identified through cited systematic reviews and meta-analyses included in this narrative review. All 26 studies were reviewed for methodological quality and relevance to the perioperative context.

Studies were identified through a structured literature search of MEDLINE and Embase. Diagnostic performance figures are reported as cited in the original publications. CUS, Compression Ultrasound; DVT, Deep Vein Thrombosis; ED, Emergency Department; ICU, Intensive Care Unit; POCUS, Point-of-Care Ultrasound; RCT, Randomized Controlled Trial; Sn, Sensitivity; Sp, Specificity.

**Supplementary Material 1**

**SANRA Checklist – Narrative Review**

**Manuscript title:**

Point-of-care ultrasound for perioperative deep vein thrombosis assessment in anesthesiology: a narrative review

**SANRA – Scale for the Assessment of Narrative Review Articles**

Each item is scored from 0 (low standard) to 2 (high standard). Total SANRA Score: 11/12

| **SANRA Item** | **Score** | **Justification** |
| --- | --- | --- |
| Justification of the article's importance for the readership | 2 | The manuscript establishes deep vein thrombosis as a major cause of preventable perioperative morbidity and highlights the relevance of point-of-care ultrasound for anesthesiologists. |
| Statement of concrete aims or formulation of questions | 2 | The review explicitly states its aim to summarize diagnostic accuracy, applicability, and practical considerations of perioperative point-of-care venous ultrasound. |
| Description of the literature search | 1 | The manuscript describes study types, populations, ultrasound protocols, and reference standards. A brief clarification of how the 26 eligible studies were identified has been added, noting that diagnostic accuracy estimates derive from previously published meta-analyses rather than original synthesis. |
| Referencing | 2 | References are current, relevant, and appropriately cited, including guidelines, consensus statements, systematic reviews, and original studies. |
| Scientific reasoning | 2 | The review critically discusses diagnostic accuracy, strengths, limitations, methodological biases, and clinical implications. |
| Appropriate presentation of data | 2 | Data are clearly synthesized in narrative form and supported by a comparative table summarizing ultrasound protocols. |
